# Effects of Exogenous Dietary Advanced Glycation End Products on the Cross-Talk Mechanisms Linking Microbiota to Metabolic Inflammation

**DOI:** 10.3390/nu12092497

**Published:** 2020-08-19

**Authors:** Raffaella Mastrocola, Debora Collotta, Giulia Gaudioso, Marie Le Berre, Alessia Sofia Cento, Gustavo Ferreira Alves, Fausto Chiazza, Roberta Verta, Ilaria Bertocchi, Friederike Manig, Michael Hellwig, Francesca Fava, Carlo Cifani, Manuela Aragno, Thomas Henle, Lokesh Joshi, Kieran Tuohy, Massimo Collino

**Affiliations:** 1 Department of Clinical and Biological Sciences, University of Turin, 10125 Turin, Italy; alessiasofia.cento@unito.it (A.S.C.); manuela.aragno@unito.it (M.A.); 2 Department of Drug Science and Technology, University of Turin, 10125 Turin, Italy; debora.collotta@unito.it (D.C.); gustavo.ferreiraalves@unito.it (G.F.A.); fausto.chiazza@uniupo.it (F.C.); roberta.verta@edu.unito.it (R.V.); 3 Fondazione Edmund Mach, 38010 San Michele all’Adige, Italy; giulia.gaudioso@fmach.it (G.G.); francesca.fava@fmach.it (F.F.); kieran.tuohy@fmach.it (K.T.); 4 Biomedical Sciences, National University of Ireland, H91 TK33 Galway, Ireland; marie.leberre@nuigalway.ie (M.L.B.); lokesh.joshi@nuigalway.ie (L.J.); 5 Department of Neuroscience, University of Turin, 10124 Turin, Italy; ilaria.bertocchi@unito.it; 6 Chair of Food Chemistry, Technische Universität Dresden, 01062 Dresden, Germany; friederike.manig@tu-dresden.de (F.M.); michael.hellwig@tu-dresden.de (M.H.); thomas.henle@tu-dresden.de (T.H.); 7 Pharmacology Unit, School of Pharmacy, University of Camerino, 62032 Camerino, Italy; carlo.cifani@unicam.it

**Keywords:** advanced glycation end products, proteins glycosylation, gut microbiota, metabolic inflammation, insulin signal pathway

## Abstract

Heat-processed diets contain high amounts of advanced glycation end products (AGEs). Here we explore the impact of an AGE-enriched diet on markers of metabolic and inflammatory disorders as well as on gut microbiota composition and plasma proteins glycosylation pattern. C57BL/6 mice were allocated into control diet (CD, *n* = 15) and AGE-enriched diet (AGE-D, *n* = 15) for 22 weeks. AGE-D was prepared replacing casein by methylglyoxal hydroimidazolone-modified casein. AGE-D evoked increased insulin and a significant reduction of GIP/GLP-1 incretins and ghrelin plasma levels, altered glucose tolerance, and impaired insulin signaling transduction in the skeletal muscle. Moreover, AGE-D modified the systemic glycosylation profile, as analyzed by lectin microarray, and increased Nε-carboxymethyllysine immunoreactivity and AGEs receptor levels in ileum and submandibular glands. These effects were associated to increased systemic levels of cytokines and impaired gut microbial composition and homeostasis. Significant correlations were recorded between changes in bacterial population and in incretins and inflammatory markers levels. Overall, our data indicates that chronic exposure to dietary AGEs lead to a significant unbalance in incretins axis, markers of metabolic inflammation, and a reshape of both the intestinal microbiota and plasma protein glycosylation profile, suggesting intriguing pathological mechanisms underlying AGEs-induced metabolic derangements.

## 1. Introduction

The progressive ageing of world population and the rapid changes in the lifestyle occurred in recent decades have contributed to the rising of chronic metabolic and inflammatory diseases [1]. In particular, nowadays nutrition is considered the main beneficial or harmful tool able to either prevent or cause metabolic inflammation (known as “metaflammation”), which is strictly related to the pathogenesis of many chronic diseases, exerting an enormous socioeconomic impact. A widely studied class of diet-derived substances with possible impact on inflammatory processes is the heterogeneous group of advanced glycation end products (AGEs). These highly reactive compounds are derived from a first reaction between a reducing sugar and the amino group of proteins and give rise, through a sequence of dehydration, cyclization, fragmentation, and oxidation reactions, to final AGE-modified proteins, which are non-degradable and functionally compromised [2]. A growing body of evidence is demonstrating the pivotal role of AGEs in several pathogenic mechanisms involving oxidative stress, inflammatory response and endothelial dysfunction, responsible for chronic diseases onset such as insulin resistance, diabetes, atherosclerosis, cardiovascular diseases, and renal dysfunction [3].

AGEs can be endogenously formed in conditions of hyperglycemia and dyslipidemia [4]. However, AGEs can also be contained in foods as a product of cooking or food processing. Indeed, particular conditions of cooking (high temperatures for long time, low level of hydration and high pH) generate large amounts of different classes of AGEs [5]. Several databases reporting AGEs quantification in the most common ingredients and popular prepared foods have been published, however, data are often contrasting and AGEs chemical characterization is limited [6,7]. Very recently, the Senate Commission on Food Safety of the German Research Foundation has published quality criteria for studies dealing with dietary glycation compounds and human health [8]. Accordingly, the best methods available for quantification of AGEs rely on chromatographic analyses, and by using these methods, a daily intake of AGEs between 25 and 75 mg was estimated [7]. Even though dietary interventions aimed to reduce AGEs intake have been demonstrated to be effective in reducing markers of oxidative stress, inflammation, and endothelial dysfunction in patients with diabetes or cardiometabolic diseases [9], it is still controversial whether, and in which amount, dietary AGEs contribute to the physiological pool of AGEs; and how they can modify systemic and tissue proteins, including their post-translational modification such as glycosylation, and affect the overall metabolism mainly in the absence of pre-existing cardiometabolic disorders. It has been estimated that a fraction of ingested AGEs, that are not absorbed and not defecated, may be metabolized intraluminally by the microbiota [10]. We have recently shown that AGEs such as N-ε-carboxymethyllysine (CML) can be metabolized by the human microbiota [11] and that E. coli is able to convert CML to mainly one metabolite, the biogenic amine N-carboxymethylcadaverine [12].

Hence, the present study aimed at investigating the effects of an AGE-enriched diet (AGE-D) on gut microbiota composition and function, as well as on the development of metabolic inflammation, focusing on the molecular pathways activated by AGEs chronic exposure at organ and tissue levels.

## 2. Materials and Methods

### 2.1. Materials

All reagents were of the highest grade of purity available and were obtained from Sigma-Aldrich (Milan, Italy). Antibodies were from Cell-Signaling Technology (Beverly, MA, USA).

### 2.2. Animals and Experimental Design

The in vivo experimental procedures here described were approved by the local Animal Use and Care Committee and the Ministry of Health (approval n°. 42/2017-PR) and are in keeping with the European Directive 2010/63/EU on the protection of animals used for scientific purposes as well as the Guide for the Care and Use of Laboratory Animals. This study was carried out using 4-weeks old C57BL/6 male mice, housed in a controlled environment at 25 ± 2 °C. Mice were randomly allocated to two experimental groups (*n* = 15 per group): mice fed with a control not-irradiated standard diet (CD) and mice fed with an AGE-enriched diet (AGE-D) for 22 weeks. AGE-D was prepared replacing casein in the CD (200 g/kg of diet) by an equal amount of modified casein where 80.5% of arginine and 41.5% of lysine were modified. The diet contained 15 μmol of MG-H1 (methylglyoxal-derived 5-hydro-5-methylimidazolone) per g of diet. MG-H1 was enriched in casein as follows: A 10% solution of sodium caseinate was mixed with MGO (40% solution, Sigma), heated for 4 h, and then lyophilized after casein precipitation. Casein and methylglyoxal were left to react in aqueous medium, whereupon casein was precipitated in order to remove methylglyoxal. Thereby, 80.5% of arginine residues were modified together with 41.5% of lysine residues, which is an unavoidable side-reaction during reaction of proteins with MGO. After lyophilization, the MGO-modified casein was used as an ingredient for the preparation of the AGEs diet (AGE-D). The fraction of arginine and lysine that had been modified was replaced in the diet by the addition of the respective amounts of free lysine and arginine, so that any effect elicited by the diet may not be due to a deficiency in these essential amino acids. This preparation contained 17.4 ± 1.5 g/kg MG-H1 as analyzed by HPLC-MS/MS after enzymatic hydrolysis [13].

All groups received water and food *ad libitum*. Body weight and food/water intake were recorded weekly, whereas fasting glucose was recorded monthly. After 22 weeks of dietary manipulation, one day before the end of the experiment, feces were collected using metabolic cages (18 h starving) after which oral glucose tolerance test (OGTT) was performed. The day after, mice (fasted for 4 h) were anesthetized using isoflurane (IsoFlo, Abbott Laboratories) and killed by cardiac exsanguination. Blood samples were collected and plasma was isolated. Submandibular salivary glands and a portion of the ileum tract of intestine were fixed in neutral buffered formalin and embedded in paraffin for histological slides preparation. The gastrocnemius muscle was isolated, frozen in liquid nitrogen and stored at −80 °C.

### 2.3. Oral Glucose Tolerance Test (OGTT)

OGTT was performed after an overnight fasting period by administering glucose (2 g/kg) by oral gavage. Once before administration and 15, 30, 60 and 120 min afterward, blood was obtained from the saphenous vein and glucose concentration was measured with a conventional glucometer (GlucoMen LX kit, Menarini Diagnostics, Italy).

### 2.4. Biochemical Analysis

The plasma lipid profile was determined by measuring the content of triglycerides (TGs), total cholesterol, high-density-lipoprotein (HDL) and low density-lipoprotein (LDL) by standard enzymatic procedures using reagent kits (Hospitex Diagnostics, Florence, Italy). Plasma insulin, ghrelin, glucose-dependent insulinotropic polypeptide (GIP), glucagon like peptide (GLP-1), plasminogen activator inhibitor (PAI)-1, IL-1β, TNF-α, IFN-γ, IL-6, IL-10 and IL-17 levels were measured by using Bio-Plex Multiplex Immunoassay System (Bio-Rad Laboratories, Hercules, CA, USA). Intestinal alkaline phosphatase (IAP) activity was detected in plasma with SensoLite pNPP Alkaline Phosphatase colorimetric assay kit (AnaSpec Inc, Fremont, CA, USA) following manufacturer’s instructions for kinetic reading.

### 2.5. Fecal Microbiota Analysis

Total genomic DNA extraction from frozen feces was carried out using QIAamp^®^ PowerFecal^®^ DNA Isolation kit (MoBio Laboratories, Inc., Carlsbad, CA, USA) and then subjected to PCR amplification by targeting 16S rRNA V3-V4 variable regions with specific bacterial primer set 341F (5′ CCTACGGGNGGCWGCAG 3′) and 806R (5′ GACTACNVGGGTWTCTAATCC 3′), as previously reported [14]. PCR products were checked by gel electrophoresis and cleaned using Agencourt AMPure XP system (Beckman Coulter, Brea, CA, USA), following manufacturer’s instructions. After 7 PCR cycles, (16S Metagenomic Sequencing Library Preparation, Illumina), Illumina adaptors were attached (Illumina Nextera XT Index Primer). Libraries were purified using Agencourt AMPure XP (Beckman) and then sequenced on an Illumina^®^ MiSeq (PE300) platform (MiSeq Control Software 2.0.5 and Real-Time Analysis software 1.16.18). Sequences obtained from Illumina sequencing were analyzed using Quantitative Insights Into Microbial Ecology (QIIME) 2.0 pipeline [15]. Percentage relative abundance of taxa from different dietary groups were compared using nonparametric Wilcoxon statistical test. Alpha and beta-diversity estimates were determined using phyloseq R Package [16]. Correlation between bacterial genera and systemic parameters in CD and AGE-D groups was performed by Spearman correlation analysis. Unidentified genera include those whose percentage sequence homology with Greengenes database was below 95% (http://greengenes.lbl.gov) [17].

### 2.6. Plasma Glycosylation Profile by Lectin Microarray

Sera from 5 mice from the CD and AGE-D were pooled and pre-cleared prior to IgG purification by centrifugation at 10,000× *g* for 15 min. IgGs were purified by protein G affinity chromatography using a protein G chromatography (Biosciences, ThermoFisher, Dublin, Ireland) following manufacturer’s instructions. Both fractions, IgGs and IgG-depleted blood, were buffered exchanged with PBS and directly fluorescently labelled with Alexa Fluor^®^ 555 (Biosciences, ThermoFisher, Dublin, Ireland) following manufacturer’s instructions, in the dark.

Lectin microarray were prepared by dilution of lectins of known specificities in phosphate buffered saline (PBS), pH 7.4, containing 1 mM concentrations of their respective haptenic sugar to ensure preservation of their binding site (Supplementary Table S1) and printed on Nexterion^®^ H (Schott, Mainz, Germany) amine-reactive, N-hydroxysuccinimide ester functionalized, hydrogel-coated glass slides using a SciFlexArrayer S3 piezoelectric spotter (Scienion, Berlin, Germany) under constant 62% (+/−2%) humidity at 20 °C. Each feature was printed with approximately 1 nL of probe using an uncoated 90 mm glass piezoelectric dispenser capillary in replicates of 6 features per probe. Eight replicate subarrays, each consisting of 52 probes in replicates of 6 features, were printed per slide. Slides were incubated in a humidity chamber overnight after printing to ensure complete conjugation. The remaining functional groups on the slide surface were then deactivated by incubation with 100 mM ethanolamine in 50 mM sodium borate, pH 8, for 1 h at room temperature. Slides were washed with PBS, pH 7.4 with 0.05% Tween-20 (PBS-T) three times for 2 min each wash and once with PBS prior to drying by centrifugation (470× *g*, 5 min). The printed lectin microarrays were stored at 4 °C with desiccant until use.as previously described [18]. Labelled IgG and D fractions were incubated on microarray slides and data extracted as described elsewhere [19]. In brief, raw intensity values were extracted from the image *.tif files using GenePix Pro v6.1.0.4 (Molecular Devices, Berkshire, UK) and a proprietary *.gal file (containing feature spot address and identity) using adaptive diameter (70–130%) circular alignment based on 230 um features and were exported as text to Excel (Version 2007, Microsoft). Local background-corrected median feature intensity data (F543median-B543) were analyzed. The median of 6 replicate spots per subarray was handled as a single data point for graphical and statistical analysis. Data were normalized to the median total intensity value of 6 replicate. Unsupervised hierarchical clustering of sample binding intensity data was performed with Hierarchical Clustering Explorer v3.0 (http://www.cs.umd.edu/hcil/hce/hce3.html). Mean total intensity, normalized by rescaling lectin binding data to 65,000 RFU, was clustered with the following parameters: no pre-filtering, complete linkage, and Euclidean distance. The significance of binding data was evaluated using a standard Student’s t test (paired, two-tailed).

### 2.7. Tissue Extracts

Gastrocnemius protein extracts were prepared as previously described [20]. Briefly, tissues were homogenized and centrifuged at 15,000× *g* for 40 min at 4 °C. Supernatants were collected and the protein content was determined using a BCA protein assay (Pierce Biotechnology Inc., Rockford, IL, USA) following manufacturer’s instructions.

### 2.8. Western Blot Analysis

About 60 µg of total proteins were loaded for Western blot experiments. Proteins were separated by 8% sodium dodecyl sulphate-polyacrylamide gel electrophoresis (SDS-PAGE) and transferred to a polyvinyldenedifluoride (PVDF) membrane, which was then incubated with primary antibodies (dilution 1:1000). The antibodies used were: rabbit anti-Ser307 IRS-1 (#2381); mouse anti-total IRS-1 (#3194); rabbit anti-Ser473 Akt (#4060); rabbit anti-total Akt (#9272); rabbit anti-Ser9 GSK–3β (#9332); and rabbit anti-total GSK–3β (#9315). Blots were then incubated with secondary antibodies conjugated with horseradish peroxidase (HRP) (dilution 1:20,000) and developed using the ECL detection system. The immunoreactive bands were analyzed by the Bio-Rad Image Lab SoftwareTM 6.0.1 and results were normalized to CD.

### 2.9. Immunohistochemistry

CML and receptor for AGEs (RAGE) immunopositivity was analyzed by immunohistochemistry on 7 μm paraffin-embedded sections of ileum and submandibular salivary glands. Slides were deparaffinized, rehydrated, and antigens were retrieved by 5 min boiling in 10 mM sodium citrate buffer, pH 6.0. After blocking, sections were incubated overnight with primary antibodies (CML, R&D, #MAB3247, dilution 1:50; RAGE, Invitrogen, #PA1-075, dilution 1:50) and subsequently for 1 h with HRP-conjugated secondary antibodies (dilution 1:200) and nuclei were counterstained with hematoxylin.

### 2.10. Statistical Analysis

All values in both the text and figures are expressed as mean ± S.E.M. for n observations. Statistical significance between CD and AGE-D values was performed by unpaired t test. OGTTs were analyzed using the area under the receiver operating characteristic (ROC) curve.

A *p* value <0.05 was considered to be statistically significant. Statistical analysis was carried out using GraphPad Prism 5.03 (GraphPad Software, La Jolla, CA, USA).

## 3. Results

### 3.1. General Parameters

Most commonly, AGEs are ingested by humans in a protein-bound form within a food matrix. Therefore, we intended to apply AGEs to mice in a protein-bound form and decided to enrich casein, an important food protein, with MG-H1, which is the predominating derivative of modification of arginine residues with methylglyoxal (MGO). HPLC-MS/MS analysis revealed that the MGO-modified casein contained 17.4 ± 1.5 g/kg of MG-H1 which, with a fraction of 20% casein in the diet, corresponds to a concentration of 3.5 g/kg or 15 µmol/g. The highest concentrations of MG-H1 in food have been found in cakes and biscuits (up to 360 mg/kg, [6]). Hence, the diet of the present study contains far more MG-H1 than may normally be ingested.

**Figure 1:**
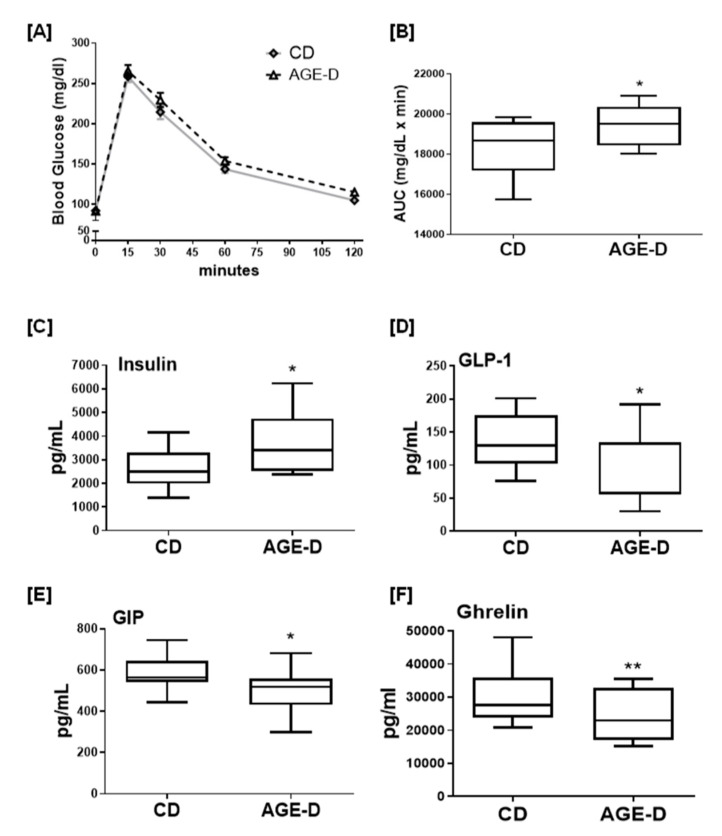
(**A**) Oral glucose tolerance test (OGTT) performed after 22 weeks of dietary manipulation on control not-irradiated standard diet (CD) and advanced glycation end products-enriched diet (AGE-D) mice. (**B**) Area under the curve showing altered OGTT in AGE-D mice indicating glucose intolerance. (**C**–**F**) Plasma levels of insulin, GLP-1, GIP, and ghrelin in CD and AGE-D mice measured by luminex suspension bead-based multiplexed Bio-Plex 3D system. Data are means ± S.E.M. (*n* = 15 per group). Statistical significance: ** *p* < 0.01, * *p* < 0.05 vs. CD.

After 22 weeks of dietary intervention, the mice exposed to AGE-D showed a robust increase in blood insulin level, associated with decreased levels of GIP and GLP-1, the two primary incretins secreted from the intestine, and ghrelin, and a significant impairment in OGTT (Figure 1), when compared to mice fed with CD. However, these effects were not associated with significant changes in body weight gain, fasting blood glucose, and lipid profile (Table 1).

### 3.2. Impact of an AGE-Enriched Diet on CML and RAGE Amounts in Salivary Glands and Intestine

**Figure 2:**
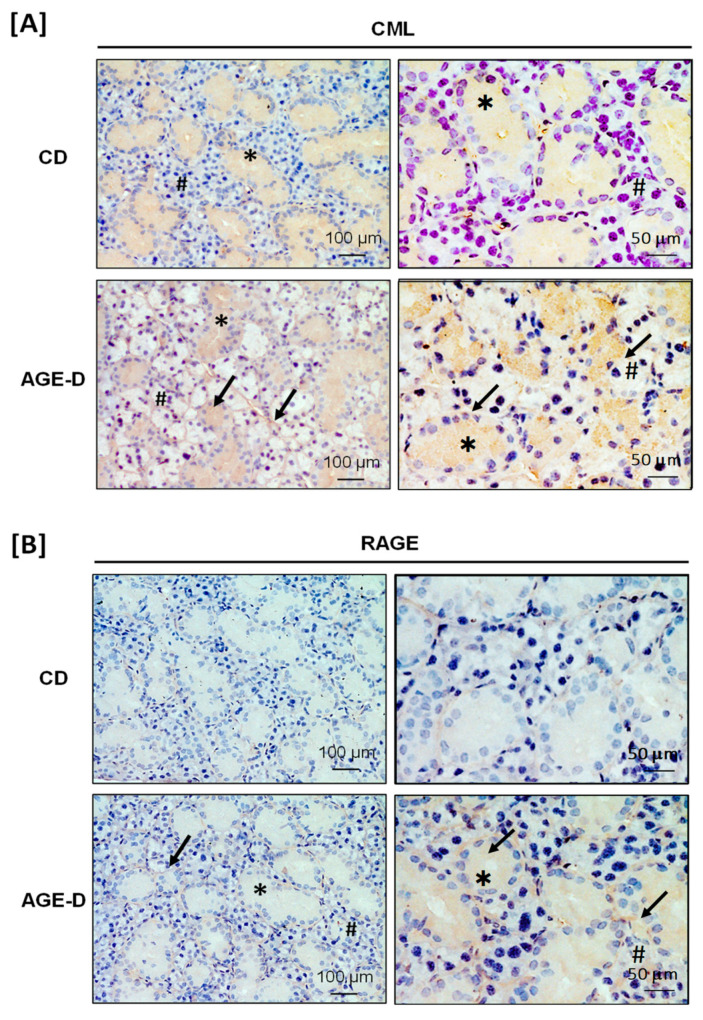
Immunohistochemistry performed on paraffin-embedded submandibular salivary glands. (**A**) Photomicrographs at 20× and 40× magnification for carboxymethyllysine (CML) immunopositivity, showing increased amounts in acini (#) and ducts (∗), as indicated by arrows, of the AGE-D mice. (**B**) Photomicrographs at 20× and 40× magnification for receptor for AGEs (RAGE) immunopositivity, which was increased in the myoepithelial and basal lamina of ducts as indicated by arrows.

**Figure 3:**
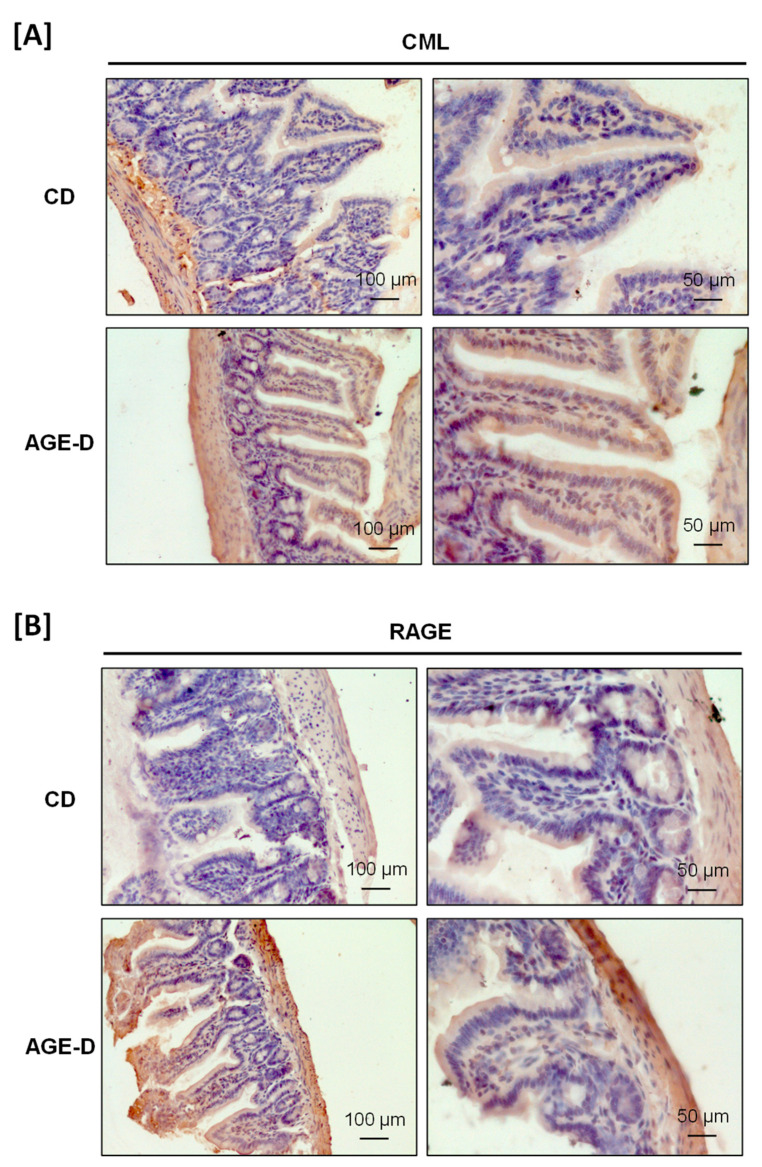
Immunohistochemistry performed on paraffin-embedded ileum portion of intestine. (**A**) Photomicrographs at 20× and 40× magnification for CML immunopositivity, showing increased amounts in villi epithelium of the AGE-D mice. (**B**) Photomicrographs at 20× and 40× magnification for RAGE immunopositivity, mostly increased in the basal membrane and muscularis mucosae.

The AGE-enriched diet evoked CML accumulation and increased RAGE expression in both submandibular salivary glands and the ileum tract of intestine detected by immunohistochemistry analysis. Specifically, in the submandibular salivary glands of AGE-D mice, we detected increased CML immunopositivity in the extracellular spaces among serous and mucous acini and in the cytoplasm of duct cells (Figure 2A), while RAGE was mainly expressed in the ducts of myoepithelial cells and basal lamina (Figure 2B), compared to the CD mice. Similarly, CML accumulation was higher in the villi epithelium of the ileum of AGE-D mice when compared to CD mice (Figure 3A) and RAGE expression was maximally expressed at the basal membrane and muscularis mucosae of AGE-D mice (Figure 3B).

### 3.3. Chronic AGEs Exposure Evokes Changes in Plasma Glycosylation

**Figure 4:**
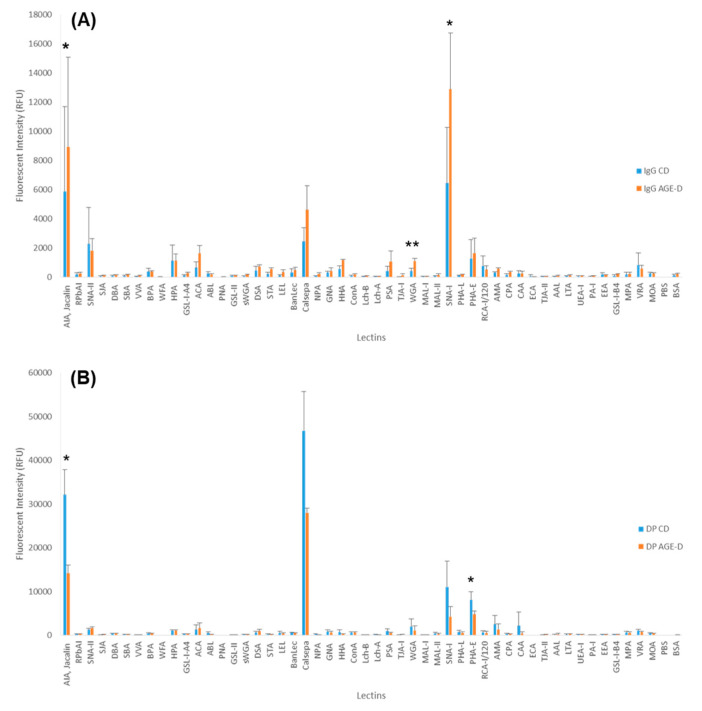
Glycosylation profiles of (**A**) immunoglobulin fractions, control diet (IgG CD) and control diet enriched in AGEs (IgG AGE-D) and (**B**) plasma glycoproteins depleted from IgG fractions, control diet (DP CD) and control diet enriched in AGEs (DP AGE-D). Bars represent the average binding intensity of fluorescently labelled samples from three technical replicate experiments and error bars represent +/− standard deviation. Statistical significance: ** *p* < 0.01, * *p* < 0.05 vs. CD, determined by two-tailed, paired Student’s t-test.

Glycosylation differences between feeding groups were investigated. Plasma samples were fractionated into two components, IgG and IgG-depleted fractions (DP), and fluorescently labelled. Glycosylation profiles of labelled fractions were compared by lectin microarray. Binding to a broad range of lectin was observed, suggesting the presence of multiple glycosylation structures (Figure 4). Similar structures were present in both fractions, albeit with diverse distribution, suggesting different glycosylation profiles between IgG and DP fractions (Figure 4A,B). The profile across CD and AGE-D in both fractions were closely comparable, with a similar glycosylation profile, despite a different distribution (Figure 4). Comparative analysis for individual lectins showed significant differences between CD and AGE-D groups. Binding on AIA, WGA, and SNA-I with labelled IgG fractions was significantly increased in the AGE-D group (Figure 4A), suggesting an increase in galactose (AIA binding), presence of N-acetylglucosamine residues (WGA binding) and sialylation which would most likely be terminal α-(2,6) linked sialic acid (WGA and SNA-I binding). On the other hand, binding on AIA and PHA-E was significantly decreased in the AGE-D group in the DP fractions (Figure 4B), implying a decrease in galactose (AIA binding) and N-linked complex type structures with β-linked Gal or Gal-β-(1,4)GlcNAc termini, with or without bisecting GlcNAc (PHA-E binding).

### 3.4. AGE-Enriched Diet Evoked Systemic Inflammatory Response

**Figure 5:**
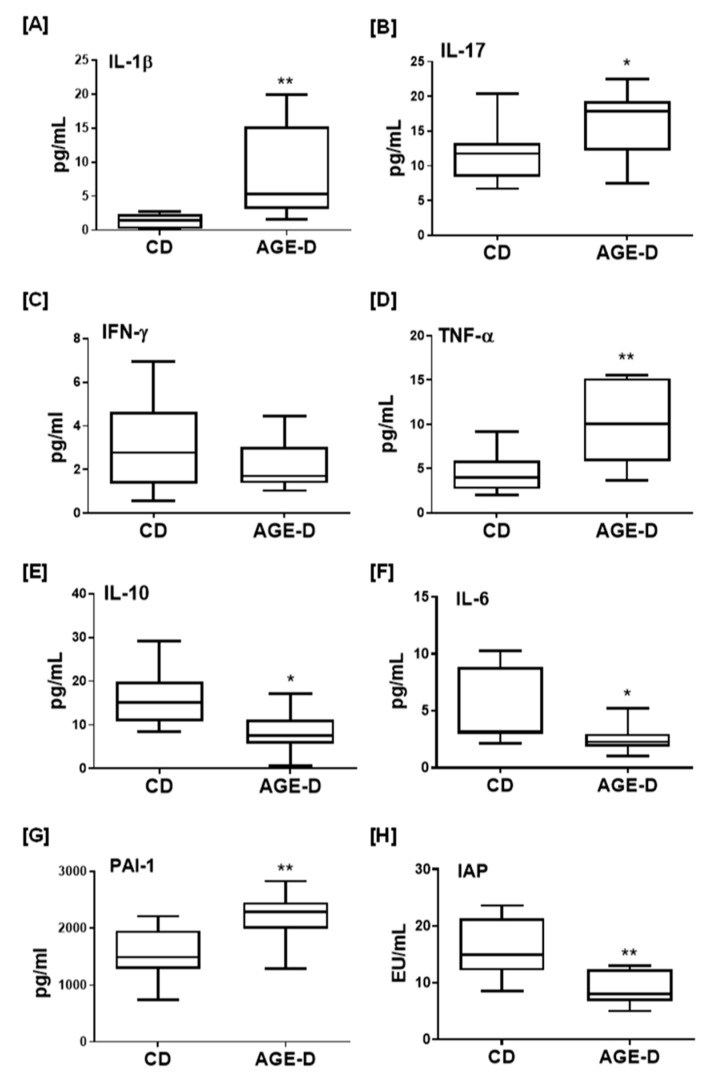
(**A**–**G**) Systemic inflammation markers evaluated in plasma of CD and AGE-D mice by multiplexed Bio-Plex 3D system, indicating increased pro-inflammatory (IL-1β, IL-17, IFN-γ, TNF-α, and PAI-1) and decreased IL-6 and IL-10. (**H**) Activity of intestinal alkaline phosphatase (IAP) evaluated in plasma by kinetic assay, indicating reduced ability of AGE-D mice to maintain microbiota homeostasis and loss of detoxifying potential in intestine. Data are means ± S.E.M. (*n* = 15 per group). Statistical significance: * *p* < 0.05, ** *p* < 0.01 vs. CD.

As shown in Figure 5, the local AGEs over-accumulation was paralleled by increased plasma levels of the pro-inflammatory cytokines IL-1β, IL-17, and TNFα and reduced levels of anti-inflammatory factors IL-6 and IL-10, with no significant effects on INF-γ. Interestingly, chronic AGE-D exposure was associated with a robust increase in blood concentrations of PAI-1, a marker of diabetes vascular complications and prothrombotic state [21], as well as with a significant decrease in the levels of IAP, a sign of impaired intestinal homeostasis and inflammation [22].

### 3.5. Chronic AGEs Exposure Altered Microbial Community Profile

**Figure 6:**
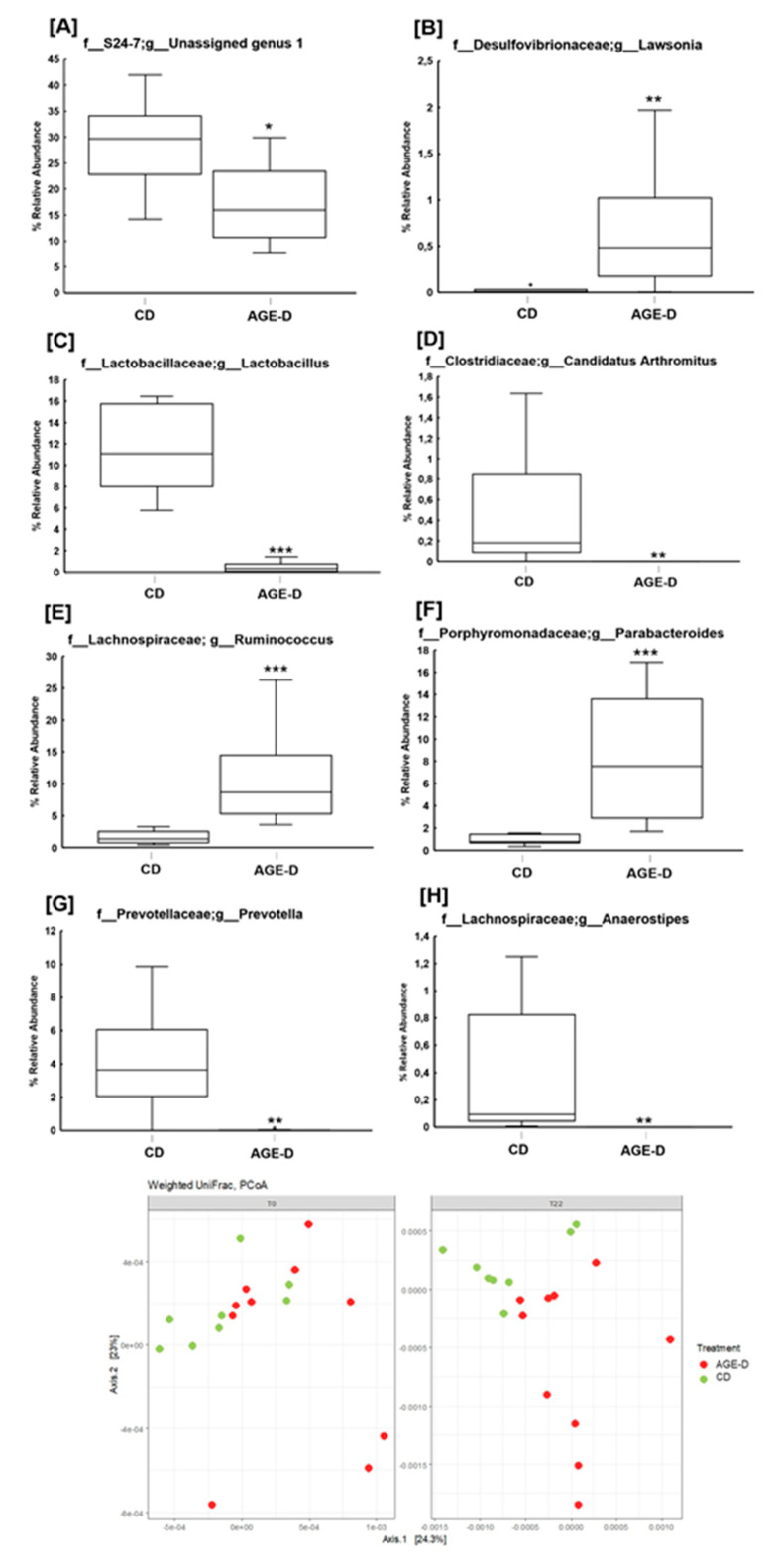
(**A**–**H**) Boxplots of percentage relative abundance of fecal microbial genera in CD (*n* = 8) and AGE-D (*n* = 10) mice at 22 weeks (T22) of dietary intervention after 16SrRNA sequencing using V3-V4 targeted primers. “Unidentified genus 1”: a genus within the Family S 24-7 which could not be assigned at a percentage sequence homology of at least 95% to any existing genera within the reference database (http://greengenes.lbl.gov). (**I**) Beta-diversity of microbial populations in CD and AGE-D at T0 and T22, according with Weighted UniFrac analysis. Statistical significance: * *p* < 0.05, ** *p* < 0.01, *** *p* < 0.001.

The analysis of microbiota revealed differences in relative abundance of fecal microbial populations (Figure 6A), with a general decreasing trend of the Bacteroidetes/Firmicutes ratio in the AGE-D group at T22 (*n* = 10) compared to CD (*n* = 8) (0.73 vs. 1.16, *p* = 0.07). No differences were observed in gut microbial composition at T0 (baseline) among the two groups. AGE-D mice differed significantly from CD in fecal microbial β-diversity at T22 weeks using Weighted UniFrac analysis (Figure 6B), but not in α-diversity (data not shown). Specifically, at family level, AGE-D mice had significantly lower S24-7 bacteria (Muribaculaceae, within the Bacteroidetes phylum, *p* < 0.05) and doubled amount of Lachnospiraceae (*p* < 0.01), in comparison to CD mice; while at the genus level, AGE-D mice had lower *Lactobacillus* (*p* < 0.001), *Prevotella* (*p* < 0.01), *Anaerostipes* (*p* < 0.01), and *Candidatus Arthromitus* (*p* < 0.01) and higher *Parabacteroides* (*p* < 0.001), *Ruminococcus* (Lachnospiraceae family, *p* < 0.001) and *Lawsonia* (*p* = 0.01) (Figure 6A).

**Figure 7:**
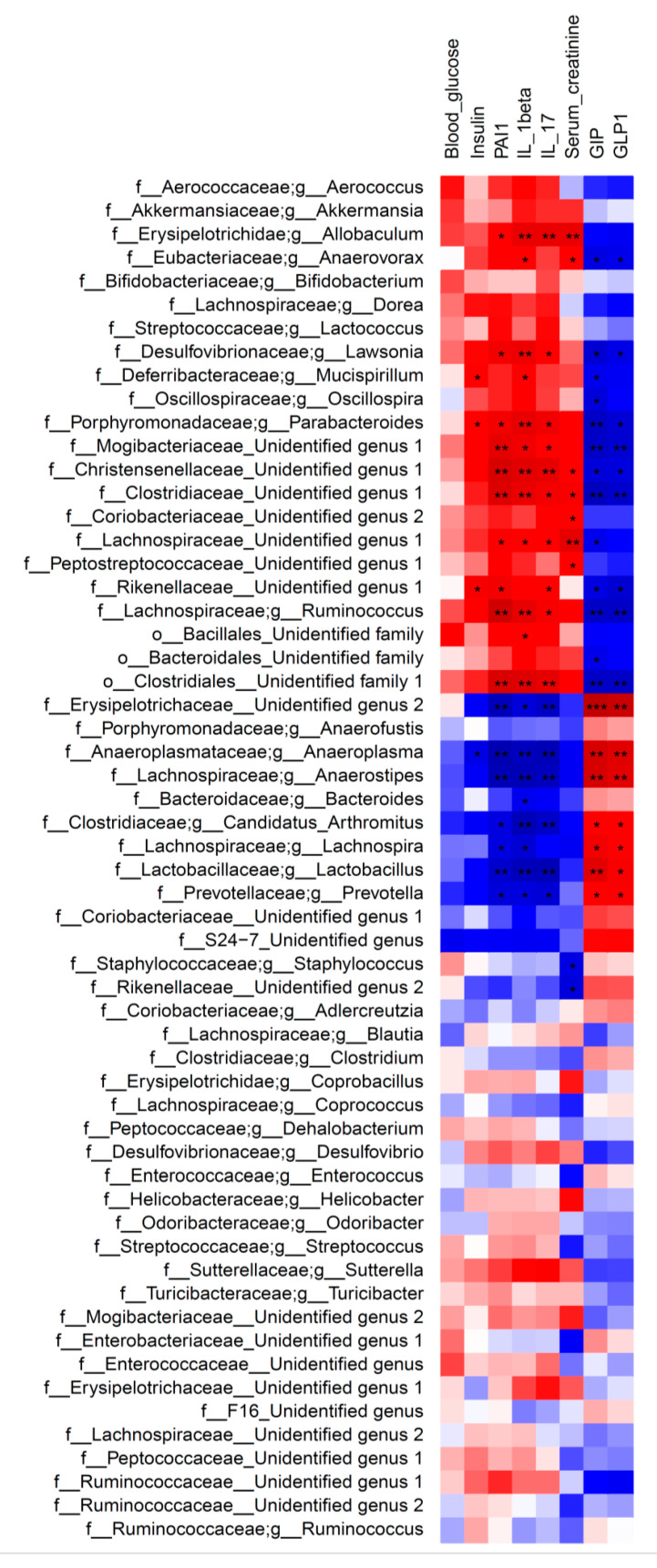
Heatmap of Spearman’s correlation between the fecal bacteria genera and systemic measurements in CD and AGE-D groups. Dark red indicates positive correlation, while dark blue represents negative correlation. Statistical significance: * *p* < 0.05, ** *p* < 0.01. Genera and families were reported as “Unidentified” when they could not be assigned to any genera/family within a given family/order at a percentage sequence homology of 95% and 90%, respectively, to existing genera and families in the reference database (http://greengenes.lbl.gov).

The heatmap of Spearman’s rank correlation coefficients in Figure 7 indicate significant correlation between relative abundance of bacterial families/genera and systemic measurements. Indeed, the overall results obtained in CD and AGE-D groups showed that Lachnospiraceae, *Parabacteroides*, *Lawsonia* and *Ruminococcus* (Lachnospiraceae family) are all positively correlated to PAI-1, IL-1β, and IL-17 levels and negatively correlated to GIP and GLP-1. Furthermore, *Prevotella*, *Lactobacillus*, *Anaerostipes*, and *Candidatus Arthromitus* have a significant positive correlation with GIP and GLP-1, and a negative correlation with systemic inflammatory blood parameters.

### 3.6. Chronic AGEs Exposure Impaired Insulin Signal Transduction in the Skeletal Muscle

**Figure 8:**
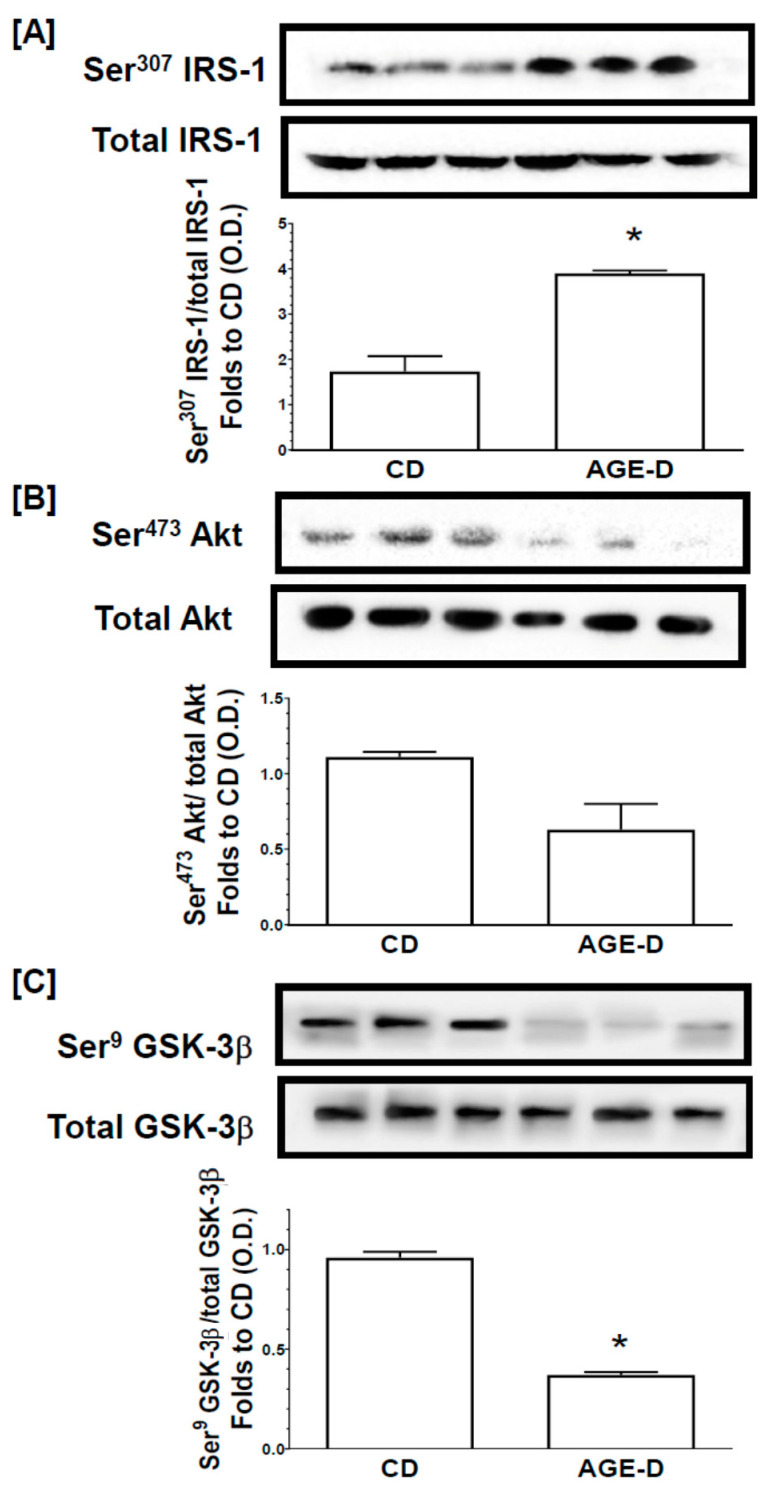
Assessment of insulin signal transduction in the gastrocnemius muscle through the Western blotting analysis of the phosphorylation rate of (**A**) IRS-1, (**B**) Akt, (**C**) GSK-3β. Histograms report the densitometric analysis represented as the ratio between phosphorylated-to-total protein amount and expressed as fold of CD value. Data are means ± S.E.M. (*n* = 15 per group). Statistical significance: * *p* < 0.05 vs. CD.

Changes in the activity of the insulin signal transduction pathway were evaluated by immunoblotting experiments on homogenates from gastrocnemius muscles (Figure 8). The AGE-D did not alter the protein expression of the insulin receptor substrate-1 (IRS-1), protein kinase B (Akt), or glycogen synthase kinase-3β (GSK-3β) in muscles, when compared to muscles from CD mice. In contrast, mice fed an AGE-enriched diet exhibited a significant increase in the degree of phosphorylation of IRS-1 on Ser307 (Figure 8A) in parallel with a reduction in the phosphorylation of downstream effectors of the insulin signaling pathway, Akt on Ser473 (Figure 8B) and a significant decrease in the phosphorylation of GSK-3β on Ser9 (Figure 8C). These alterations in protein phosphorylation, and hence activation status of the respective proteins are suggestive of an impairment in insulin signaling evoked by the AGE-enriched diet.

## 4. Discussion

In the present study we reported for the first time that the enrichment of a standard diet with MG-H1, a common dietary AGEs found in highly processed foods [23], is sufficient to evoke AGEs tissue accumulation. These effects were associated with a pro-inflammatory state and changes in early markers of dysmetabolism, more likely through alterations in microbiota homeostasis. We used a non-irradiated standard diet enriched in only MG-H1 to investigate the effective causal contribution of a well-characterized AGE in metabolic derangements, excluding the effect of other factors such as food processing products or alternative sources of AGEs. Interestingly, the significant changes recorded in the blood levels of key master hormonal regulators of metabolism were associated with local impairment of the insulin signaling pathway, which is a crucial regulator of glucose transportation, glycogen synthesis and glycolysis. However, these modifications were not associated with changes in body weight gain, fasting blood glucose, and lipid profile, suggestive of a condition of early metabolic derangement. Longer kinetics of dietary manipulation and/or more severe dietary insult would be requested to confirm the clinical relevance of AGEs exposure in vivo. Our findings are in accordance with previously in vitro studies demonstrating that cellular exposure to AGEs resulted in impaired secretion and activity of GIP and GLP-1, the two primary incretin hormones [24,25] and increased expression of dipeptidyl peptidase-4 (DPP-4), the main enzyme degrading incretins [26,27]. Therefore, it is conceivable that dietary AGEs impair the effects of incretins, further promoting the development of metabolic disorders. The AGE-D-induced reduction in incretin levels along with the well-known AGEs ability to induce activation of inflammatory transcription factors through interaction with RAGE [28], may account for the here recorded increase in blood concentrations of pro-inflammatory cytokines and PAI-1. In fact, GLP-1 plays a vital role in modulating cytokines function and their production by CD4+ T cells via GLP-1 receptors [29] and, in keeping with our findings, the GLP-1 analogue exenatide has been demonstrated to reduce the levels of IL-1β, IL-17 and TNF-α in human islet supernatants [30]. Interestingly, among the panel of cytokines we tested, IL-10 and IL-6 were the ones whose systemic concentrations were significantly reduced following AGE-D. IL-6 is a pro-inflammatory cytokine. However, at the local level, it may exert several anti-inflammatory actions, including downregulation of IFN-γ, IL-1β, and TNF-α [31]. Most notably, GLP-1 secretion is regulated by IL-6 [32]; thus, offering a further insight on the molecular mechanism linking dietary AGEs exposure to impairment in incretin levels. Ghrelin signaling is another key mediator linking nutrient-sensing signals with insulin resistance, and ablation of ghrelin has been reported to worsen diet-induced insulin resistance and adipose inflammation [33]. As previously documented [34,35], ghrelin may contribute to the physiological anti-AGEs system, counteracting the deleterious effects exerted by AGEs on vascular endothelium. The decrease in ghrelin concentration following AGE-D in this study was paralleled by a massive increase in the plasma levels of PAI-1, a key regulator of vascular remodeling, involved in various thrombotic diseases such as deep vein thrombosis, ischemic heart disease and diabetic vascular complications. AGEs may induce a RAGE-mediated functional synthesis of PAI-1 in human microvascular endothelial cells [36], and blood AGEs levels in either non-diabetic and diabetic populations are one of the most important independent determinants of PAI-1 [37,38]. We may therefore speculate that the changes in ghrelin and PAI-1 concentrations induced by AGE-D may contribute to early cellular senescence and impaired vascular integrity. However, the lack of investigation on the effects of the AGE-D at vascular level on thrombogenic and anti-fibrinolytic changes, does not allow us to confirm a significant impact of AGE-D on cardiovascular risk factors in our experimental model.

Our study also offers an interesting insight on the relative contribution of exogenous dietary AGEs to the impairment of metabolic homeostasis. Maillard reaction, commonly known as protein glycation, normally occurs in vivo as well as during the preparation of foods at high temperatures. Here, AGEs diet was enriched in MG-H1, which is one of the most important Maillard reaction product identified and quantified in food and biological matrices [23], and the most abundant in body fluids of diabetes patients [39,40]. The AGE-D differed from the control diet only for the presence of MG-H1 instead of a part of the arginine residues in the casein; thus, indicating that all the systemic and tissue alterations here recorded have to be related to this dietary modification.

Several cross-sectional and intervention studies have shown positive correlations between AGEs intake and their circulating levels, as measured by food databases [2,6,41]. Isocaloric restrictions of dietary AGEs have been shown to decrease circulating AGEs levels and inflammatory biomarkers, and to improve endothelial dysfunction [42]. However, the mechanisms linking dietary AGEs exposure to their absorption and their effective bioavailability, are still largely unknown. Here we recorded an important local accumulation of AGEs and overexpression of RAGE not only in the ileum tract of intestine but also in submandibular salivary glands; thus, confirming the potential correlation between dietary AGEs and periodontal pathology, recently suggested by several studies [43]. Interestingly, the specific Maillard reaction product we detected was CML, which is a chemical entity different from MG-H1 and not included in the modified diet. These results imply that AGEs found in salivary glands originate, at least in part, from blood. This hypothesis has been recently confirmed in an intervention study on healthy subjects exposed to diets with different amount and quality of AGEs [13]. Nevertheless, the specific mechanisms of transports of AGEs from blood to saliva remains to be elucidated. Interestingly, CML fecal excretion does not exceed the 50% [44], suggesting that some of the ingested AGEs are neither absorbed nor defecated and could be metabolized intraluminally by the microbiota. Thus, it is likely that protein-bound dietary AGEs are processed at the consumption of an MG-H1-enriched diet, resulting in accumulation of a different class of AGEs, such as CML, in both proximal (ileum) and distal (salivary glands) organs/tissues. The intestinal AGEs processing is due to specific microorganisms and local AGEs accumulation may affect gut microbiota through negative selection for direct toxic effects, or positive selection favoring bacterial species that use AGEs as source of energy [45]. Here, for the first time, we demonstrated that a diet enrichment with a single AGEs is sufficient to induce significant changes in the microbiota composition. Notably, the MG-H1 enriched diet here used was neither heated nor irradiated; thus, offering an appropriate experimental approach to detect the impact of AGEs on gut microbiota. Indeed, many contradictory data have been reported on the effect of heated foods on microbiota due to the heterogeneity of compounds that are formed during thermal treatment [46,47,48]. Our results showed marked differences in gut microbiota population of AGE-D mice, characterized by a depletion of commensal bacteria such as S24-7, *Candidatus Arthromitus* and *Anaerostipes*. Among them, *Candidatus Arthromitus* plays a key role in mouse intestinal immune function control and its downregulation may be associated with intestinal inflammatory imbalance [49]. In addition, AGE-D mice showed a decrease of a butyrate-producing bacterial genus, *Anaerostipes*, that is inversely related to inflammation and insulin resistance, since butyrate is reported as one of the most important short-chain fatty acids (SCFAs) in the maintenance of colonic health [50]. Moreover, we also found an increase of *Parabacteroides*, *Ruminococcus* (Lachnospiraceae family) and *Lawsonia* in the AGE-D group. An abnormal increase in Lachnospiraceae has been recently proposed as one of the factors involved in metabolic diseases such as diabetes and obesity [50], but the mechanism through which these bacteria affect these conditions is still unclear. It has been proposed that members of Lachnospiraceae may be involved in intestinal lipopolysaccharide translocation in blood, thus becoming one of the causes of the inflammatory processes which characterize these metabolic diseases [51]. Our results support previous studies where *Lactobacillus* spp. ameliorate Type 2 diabetes by acting on GLP-1 mechanism [52]. *Prevotella* is a dietary fiber fermenter bacterium, known to increase after a high fiber intake [53] and to produce SCFAs [54], which affect satiety regulation and glucose metabolism by increasing GLP-1 and other gut hormones production [55]. This mechanism may provide a link between *Prevotella* reduction in AGE-D mice and incretin production. Diet induced shifts in gut microbial population by modulating SCFAs production: we can speculate that AGE-enriched diet may affect incretins production by a microbiota-driven mechanism, in which *Prevotella* and other fiber-fermenting and SCFAs-producing bacteria are decreased. The rise of *Lawsonia* abundance was previously observed in diabetic mice fed with high-fat chow and was seen to decrease after metformin treatment, which normally acts by increasing GLP-1 production and glucose utilization [56,57,58]. Since AGEs seem to reduce GLP-1 levels as described above, we speculated that *Lawsonia* increase in AGE-D mice may be caused by incretins unbalance and systemic changes induced by MG-H1. Many of the microbial alterations observed in AGE-D group were significantly related to incretins and inflammatory markers levels and have been associated in previous studies with obesogenic and/or diabetogenic environments. Interestingly, compared to CD, the AGE-D was not characterized by a higher fat content, and mice fed with AGE-D did not show an increase in body weight gain and feeding behavior. This suggests that the simple enrichment of MG-H1 in the diet caused a reshaping of the microbiota that is normally observed in high-fat diets or in the presence of inflammatory conditions such as diabetes. Our results showed that systemic unbalance caused by AGEs enrichment in diet, mainly in the pro-inflammatory profile, incretins axis, and glucose control, induced significant changes in gut microbial populations. Furthermore, these shifts resemble what has previously been seen in obesity, diabetes, and metabolic disorders.

Moreover, our glycomic analysis using lectin microarray indicates for the first time that even one specific class of AGEs contained in food (i.e., MG-H1) can trigger modification of the post-translational glycosylation profile of peripheral blood proteins. Alteration in the glycosylation profile of plasma and blood cell surface proteins, including IgGs, can impact on their conformation and functionality; thus, interfering with key physiological processes. The observed alteration of blood protein glycosylation is most likely associated with changes in the plasma level of acute phase proteins, with circulating cytokines and hormones [59], diet, and lifestyles known to affect glycosylation level [60]. IgG glycosylation is known to be altered by environmental and in vivo status, and, in turn, to influence the immune response, acting therefore as a potential dynamic biomarker for disease or therapeutics [61]. Significant increase in galactosylation and IgG sialylation, most likely α-(2,6)-linked, were observed in mice fed with AGE-enriched diet compared to the control group. Level of sialylation is known to correlate to level of galactosylation [62]. Our feeding study showed an increase in circulatory pro-inflammatory cytokines; therefore, suggesting an activation of inflammation transcription factors. Decrease in sialylation and galactosylation is often associated with poor metabolic health [63,64] and with chronic inflammatory disease [65]. Whereas the opposite has been shown to be linked with anti-inflammatory response, with α-(2,6)-linked sialylation playing a key role in mediating the response [66,67]. However, in agreement with the cytokine profile here evoked by the AGE-D, it has been reported that the sialylated IgG fraction reduces phagocytosis by monocytes and induces a switch of the cytokine profile from IL-6/IL-8 to TNF-α/IL-1β [68]. We could thus hypothesize that in the acute body response to the AGE-D, the IgG glycosylation is altered in an attempt to counteract or attenuate the effect of the circulatory proinflammatory cytokines.

Our study has several limitations. First, the dietary content of MG-H1, which is far more than the amount that may normally be ingested. In addition, no significant changes in systemic lipid and glucose profile were recorded, despite the significant changes in the blood levels of key master hormonal regulators of metabolism; thus, suggesting that longer kinetics of dietary manipulation and/or more severe dietary insult are requested to obtain clinically relevant metabolic derangements. Our study shows that chronic MG-H1 exposure results in local (submandibular glands, ileum, and skeletal muscle) and systemic toxicity. However further organs and tissues involved in cardiometabolic derangements, including adipose tissue, liver, and vascular endothelium, should be analyzed to offer a better elucidation of the MG-H1 on-target toxicity.

## 5. Conclusions

In conclusion, the present work provides original findings linking the presence of a specific AGEs in the diet to alterations in the microbiota homeostasis and the related incretins axis that lead to a systemic pro-inflammatory profile responsible for compromised glucose control and endothelial dysfunction. Overall, these findings help to elucidate the pivotal role of AGEs as a striking link between modern diet and health, moving from correlation toward causation. Further experimental and clinical studies are needed to highlight the importance of specific AGEs in human metabolism and disease, as well as data revealing how AGEs can elicit specific signaling functions, in the perspective to prevent the progression of diet-related metabolic derangements.

## Figures and Tables

**Table 1:** Effects on mice body weight and systemic lipid/glucose profile at 22 weeks of the AGE-enriched diet (AGE-D) in comparison to the control diet (CD).

	CD	AGE-D
Body weight (g)	29.3 ± 2.5	27.5 ± 2.1
Body weight gain	0.83 ± 2.30	0.62 ± 2.23
Food intake (g/day)	3.60 ± 0.36	3.30 ± 0.25
Water intake (mL/day)	4.81 ± 0.15	4.82 ± 0.20
Caloric intake (cal/day)	13.9 ± 1.4	11.9 ± 1.0
Triglyceride (mg/dL)	75 ± 5	79 ± 2
Total cholesterol (mg/dL)	110 ± 6	118 ± 4
HDL cholesterol (mg/dL)	63 ± 10	60 ± 3
Glucose (mg/dL)	86 ± 3	89 ± 4

Data are means ± S.E.M. (*n* = 15). No statistically significant differences were recorded for the tested marker.

## Supplementary Materials

The following are available online at https://www.mdpi.com/2072-6643/12/9/2497/s1, Table S1: lectin microarray composition list and lectin specificities
