# Supplementary material for: Effects of Exogenous Dietary Advanced Glycation End Products on the Cross-Talk Mechanisms Linking Microbiota to Metabolic Inflammation
Source: Nutrients. 2020 Aug 19;12(9):2497. doi: 10.3390/nu12092497 (PMC7551182; doi:10.3390/nu12092497)
Supplement: Supplementary file 1 [file nutrients-12-02497-s001.pdf]

Supplementary Table S1. Lectin microarray composition list and lectin specificities

No	Lectin	Common name	Type	Specificity
1	AIA, Jac	Jack fruit lectin	Plant	Gal (sialylation tolerant)
2	RPbAI	Black locust lectin	Plant	Gal, GalNAc
3	SNA-II	Sambucus lectin-II	Plant	Gal/GalNAc
4	SJA	Pagoda tree lectin	Plant	β-GalNAc
5	DBA	Horse gram lectin	Plant	GalNAc
6	SBA	Soy bean lectin	Plant	GalNAc
7	VVA	Hairy vetch lectin	Plant	GalNAc
8	BPA	Camel's foot tree lectin	Plant	GalNAc/Gal
9	WFA	Japanese wisteria lectin	Plant	GalNAc/sulfated GalNAc
10	HPA	Garden snail lectin	Animal	α-GalNAc
11	GSL-I-A4	Griffonia lectin-I A4	Plant	GalNAc
12	ACA	Amaranthin	Plant	Sialylated/Gal-β-(1,3)-GalNAc
13	ABL	Edible mushroom lectin	Fungus	Gal-β-(1,3)-GalNAc, GlcNAc
14	PNA	Peanut lectin	Plant	Gal-β-(1,3)-GalNAc
15	GSL-II	Griffonia/Bandeiraea lectin-II	Plant	GlcNAc
16	sWGA	Succinyl WGA	Plant	GlcNAc
17	DSA	Jimson weed lectin	Plant	GlcNAc
18	STA	Potato lectin	Plant	GlcNAc oligomers
19	LEL	Tomato lectin	Plant	GlcNAc-β-(1,4)-GlcNAc
20	BanLec	Banana lectin	Plant	Man/Glc in α-(1,3) linkage; laminaribose Glc-β-(1,3)-Glc
21	Calsepa	Bindweed lectin	Plant	Man/Maltose
22	NPA	Daffodil lectin	Plant	α-(1,6)-Man
23	GNA	Snowdrop lectin	Plant	Man-α(1,3)-
24	HHA	Amaryllis agglutinin	Plant	Man-α(1,3)-Man-α(1,6)-
25	ConA	Jack bean lectin	Plant	Man, Glc, GlcNAc
26	Lch-B	Lentil isolectin B	Plant	Man, fucose dependent
27	Lch-A	Lentil isolectin A	Plant	Man, fucose dependent
28	PSA	Pea lectin	Plant	Man, fucose dependent
29	TJA-I	TJA One	Plant	Sialic acid-α-(2,6)-Gal(NAc)
30	WGA	Wheat germ agglutinin	Plant	NeuAc/GlcNAc
31	MAL-I	Maackia agglutinin I	Plant	Siaα(2,3)Galβ(1,4)GlcNAc; SO_4-3-Galβ(1,4)GlcNAc
32	MAL-II	Maackia agglutinin II	Plant	Siaα(2,3)Galβ(1,3)(±Siaα(2,6))GalNAc; SO_4-3-Galβ
33	SNA-I	Sambucus lectin-I	Plant	Sialic acid-α-(2,6)-Gal(NAc)
34	PHA-L	Kidney bean leukoagglutinin	Plant	tri-/tetra-antennary β-Gal/Gal-β-(1,4)-GlcNAc
35	PHA-E	Kidney bean erythroagglutinin	Plant	biantennary, bisecting GlcNAc,β-Gal/Gal-β-(1,4-)GlcNAc
36	RCA-I/120	Castor bean lectin I	Plant	Gal-β-(1,4)-GlcNAc
37	AMA	Lords and Ladies agglutinin	Plant	Gal-β-(1,4)-GlcNAc
38	CPA	Chickpea lectin	Plant	Complex glycopeptides
39	CAA	Pea tree lectin	Plant	Gal-β-(1,4)-GlcNAc
40	ECA	Cocks comb/coral tree lectin	Plant	Gal-β-(1,4)-GlcNAc oligomers
41	TJA-II	TJA Two	Plant	Fuc-α(1,2)Gal(NAc)-β(1,4)
42	AAL	Orange peel fungus lectin	Fungus	α-(1,6 and 1,3)-linked Fuc
43	LTA	Lotus lectin	Plant	α-(1,3)-linked Fuc
44	UEA-I	Gorse lectin-I	Plant	α-(1,2)-linked Fuc
45	PA-I	Pseudomonas lectin	Bacteria	α-Gal, Gal derivatives
46	EEA	Spindle tree lectin	Plant	α-Gal
47	GSL-I-B4	Griffonia/Bandeiraea lectin-I	Plant	α-Gal
48	MPA	Osage orange lectin	Plant	α-Gal
49	VRA	Mung Bean Lectin	Plant	α-Gal
50	MOA	Fairy ring mushroom lectin	Fungus	α-Gal
51	PBS	N/A	N/A	N/A
52	BSA	N/A	Animal	N/A
